# Supplementary material for: Structural and functional asymmetry of the neonatal cerebral cortex
Source: Nat Hum Behav. Author manuscript; Available in PMC 2026 Mar 21. (PMC7618908; doi:10.1038/s41562-023-01542-8)
Supplement: Reporting Data [file EMS212917-supplement-Reporting_Data.pdf]

nature portfolio

Corresponding author(s): Dr. Logan Z. J. Williams, Dr. Emma C. Robinson

Last updated by author(s): 18/01/2022

Reporting Summary

Nature Portfolio wishes to improve the reproducibility of the work that we publish. This form provides structure for consistency and transparency in reporting. For further information on Nature Portfolio policies, see our Editorial Policies and the Editorial Policy Checklist.

Statistics

For all statistical analyses, confirm that the following items are present in the figure legend, table legend, main text, or Methods section.

n/a	Confirmed	
☐	☒	The exact sample size (*n*) for each experimental group/condition, given as a discrete number and unit of measurement
☐	☒	A statement on whether measurements were taken from distinct samples or whether the same sample was measured repeatedly
☐	☒	The statistical test(s) used AND whether they are one- or two-sided<br>*Only common tests should be described solely by name; describe more complex techniques in the Methods section.*
☐	☒	A description of all covariates tested
☐	☒	A description of any assumptions or corrections, such as tests of normality and adjustment for multiple comparisons
☐	☒	A full description of the statistical parameters including central tendency (e.g. means) or other basic estimates (e.g. regression coefficient) AND variation (e.g. standard deviation) or associated estimates of uncertainty (e.g. confidence intervals)
☐	☒	For null hypothesis testing, the test statistic (e.g. *F*, *t*, *r*) with confidence intervals, effect sizes, degrees of freedom and *P* value noted<br>*Give P values as exact values whenever suitable.*
☒	☐	For Bayesian analysis, information on the choice of priors and Markov chain Monte Carlo settings
☒	☐	For hierarchical and complex designs, identification of the appropriate level for tests and full reporting of outcomes
☐	☒	Estimates of effect sizes (e.g. Cohen's *d*, Pearson's *r*), indicating how they were calculated

Our web collection on statistics for biologists contains articles on many of the points above.

Software and code

Policy information about availability of computer code

Data collection	No software was used for data collection for this study
Data analysis	This study utilised the following open software and code: surface atlas creation (without symmetrisation): https://github.com/jelenabozek/SurfaceAtlasConstruction; dHCP structural pipeline: https://github.com/BioMedIA/dhcp-structural-pipeline and dHCP functional pipeline: https://git.fmrib.ox.ac.uk/seanf/dhcp-neonatal-fmri-pipeline/-/tree/master; FSL version 6.0.3 (for MIGP and MELODIC): https://fsl.fmrib.ox.ac.uk/fsl/fslwiki/; symmetrising resting-state timeseries: https://git.fmrib.ox.ac.uk/seanf/asymmetry-analysis; surface registration, metric and anatomical mesh resampling: https://github.com/ecr05/dHCP_template_alignment; MSM: https://github.com/ecr05/MSM_HOCR/releases; dHCP MSM configuration file: https://github.com/ecr05/dHCP_template_alignment/blob/master/configs/config_subject_to_40_week_template_3rd_release; HCP MSM configuration file optimised for sulcal depth: https://github.com/metrics-lab/CorticalAsymmetry/blob/main/config_standard_MSMstrain_HCP_CorticalAsymmetry; PALM version alpha119: https://github.com/andersonwinkler/PALM; INTERGROWTH-21 growth curves: http://intergrowth21.ndog.ox.ac.uk/; Connectome Workbench version 1.5: https://www.humanconnectome.org/software/connectome-workbench; Pingouin Python package version 0.5.3: https://pingouin-stats.org/. Code used to perform image processing and asymmetry analyses is available at \url{https://github.com/metrics-lab/CorticalAsymmetry}

For manuscripts utilizing custom algorithms or software that are central to the research but not yet described in published literature, software must be made available to editors and reviewers. We strongly encourage code deposition in a community repository (e.g. GitHub). See the Nature Portfolio guidelines for submitting code & software for further information.

Data

Policy information about availability of data

All manuscripts must include a data availability statement. This statement should provide the following information, where applicable:

- Accession codes, unique identifiers, or web links for publicly available datasets
- A description of any restrictions on data availability
- For clinical datasets or third party data, please ensure that the statement adheres to our policy

The images used to produce the figures presented here are available as scenes through https://balsa.wustl.edu/study/2xrBN. UKB asymmetry summary measures reported in Sha et al. sha2021handedness are available at https://archive.mpi.nl/mpi/islandora/object/mpi:1839_24c1553d_3ee8_4879_8877_79ca19a0ac6a?asOfDateTime=2021-11-02T14:30:48.830Z. The following templates are publicly available: dhcpSym spatiotemporal cortical surface atlas: https://brain-development.org/brain-atlases/atlases-from-the-dhcp-project/cortical-surface-template/; HCP sulcal depth template: https://github.com/Washington-University/HCPpipelines/tree/master/global/templates/standard_mesh_atlases; deformations between HCP fs_LR and FreeSurfer fsaverage space: https://github.com/Washington-University/HCPpipelines/tree/master/global/templates/standard_mesh_atlases/resample_fsaverage. Demographic data for the dHCP are available at https://github.com/BioMedIA/dHCP-release-notes/blob/master/supplementary_files/combined.tsv. HCP imaging data (all publicly available) were downloaded through ConnectomeDB: https://db.humanconnectome.org/app/template/Login.vm. dHCP imaging data (all publicly available) were downloaded through the official release website: https://data.developingconnectome.org/app/template/Login.vm.

Field-specific reporting

Please select the one below that is the best fit for your research. If you are not sure, read the appropriate sections before making your selection.

☒ Life sciences ☐ Behavioural & social sciences ☐ Ecological, evolutionary & environmental sciences

For a reference copy of the document with all sections, see nature.com/documents/nr-reporting-summary-flat.pdf

Life sciences study design

All studies must disclose on these points even when the disclosure is negative.

Sample size	Developing Human Connectome Project: The sample size of this project was determined by first assessing with dHCP participants had both structural and functional MRI scans. After this initial sample size, participants were included if they met eligibility criteria specific for this study (gestational age at birth and postmenstrual age at scan). Incidental findings with possible clinical significance but unlikely analysis significance were permissible.<br>Human Connectome Project - Young Adult: Imaging data from 1100 subjects were used, which is the number of subjects with structural imaging data available from ConnectomeDB.<br>Rationale for sample size: no statistical tests were used to determine sample sizes for this study. However, these two studies represent the largest and highest quality datasets available for neonates (dHCP) and healthy young adults (HCP-YA). All datasets used here have already been shown to be sufficiently high powered to offer important insights into cortical organisation.
Data exclusions	dHCP: Of the eligible participants, data were excluded if any participant had incidental findings with clinical significance e.g. destructive white matter lesions
Replication	This study replicated many of the structural and functional asymmetries that have been previously described in neonates and infants
Randomization	No interventions or treatments were allocated in this study, so randomisation was not required.
Blinding	No interventions or treatments were allocated in this study, so blinding was not required.

Reporting for specific materials, systems and methods

We require information from authors about some types of materials, experimental systems and methods used in many studies. Here, indicate whether each material, system or method listed is relevant to your study. If you are not sure if a list item applies to your research, read the appropriate section before selecting a response.

Materials & experimental systems

n/a	Involved in the study
☒	☐ Antibodies
☒	☐ Eukaryotic cell lines
☒	☐ Palaeontology and archaeology
☒	☐ Animals and other organisms
☐	☒ Human research participants
☒	☐ Clinical data
☒	☐ Dual use research of concern

Methods

n/a	Involved in the study
☒	☐ ChIP-seq
☒	☐ Flow cytometry
☐	☒ MRI-based neuroimaging

Human research participants

Policy information about studies involving human research participants

Population characteristics	dHCP: Data from 442 healthy term-born neonates (200 females), and 103 preterm-born neonates (48 females) were included. Term neonates were born at 39.9±1.2 weeks' GA, and scanned at 41.2±1.7 weeks' PMA. Preterm neonates were born at 32.1 ±3.5 weeks' GA, and scanned at 40.8±2.1 weeks' PMA.<br>HCP-YA: We included 1110 healthy young adults (605 biological females), who were scanned at 28.8±3.7 years.
Recruitment	dHCP: Healthy term and preterm neonates were recruited as part of the Developing Human Connectome Project, and were recruited with the maternity and neonatal units in St. Thomas Hospital, London, UK. All neonates needed to be clinically stable in order to be scanned, which was mostly relevant for preterm neonates. Consequently, preterm neonates tended to be born and scanned later (many of the preterm neonates recruited were moderate-to-late preterm). As outlined in the manuscript, we did not investigate how the degree of preterm birth affects cortical asymmetry and believe that this is an important consideration when interpreting results.<br><br>HCP-YA: These participants were recruited from ~300 families of twins and their non-twin siblings, provided written informed consent prior to imaging and were scanned at Washington University in St. Louis between 2012 and 2015. The inclusion criteria for the HCP-YA was very strict - importantly, preterm birth was an important exclusion criterion. Therefore, we are confident that our results are reflective of asymmetries in healthy young adults.
Ethics oversight	dHCP: This study was approved by the London - Riverside Research Ethics Committee of the Health Research Agency (REC:14/Lo/1169)<br>HCP-YA: This study was approved by the internal review board of Washington University in St. Louis (IRB #201204036)

Note that full information on the approval of the study protocol must also be provided in the manuscript.

Magnetic resonance imaging

Experimental design

Design type	dHCP: Structural MRI and resting-state functional MRI images were acquired and analysed<br>HCP-YA: Only structural MRI were analysed, although structural, functional (task and resting state), and diffusion images were acquired.
Design specifications	dHCP: All data were acquired in a single scan session<br>HCP-YA: All images were acquired over two sessions
Behavioral performance measures	dHCP + HCP-YA: No behavioural measures were analysed as part of this study

Acquisition

Imaging type(s)	Structural, resting-state functional
Field strength	3 Tesla
Sequence & imaging parameters	dHCP: T2-weighted scans were acquired with a repetition time/echo time (TR/TE) of 12s/156ms, SENSE=2.11/2.58 (axial/sagittal) with in-plane resolution of 0.8x0.8mm, slice thickness of 1.6 and overlap of 0.8mm. Images were motion corrected and super-resolution reconstructed resulting in 0.5mm isotropic resolution. fMRI scans were acquired over 15 minutes 3 seconds (2300 volumes) using a multislice gradient-echo echo planar imaging sequence with multiband excitation (multiband factor 9). TR/TE was 392ms/38ms milliseconds, flip angle was 34°, and the acquired spatial resolution was 2.15mm isotropic.<br>HCP-YA: Full details regarding image acquisition have been previously reported. Images were acquired on a customised 3-Tesla Siemens Skyra (Siemens AG, Erlangen, Germany) using a 32-channel head coil. T1w scans (3D MPRAGE) were acquired with a TR/TE of 2400 ms/2.14 ms, and inversion time of 1000 ms, and flip angle of 8°. T2w scans (Siemens SPACE) were acquired with a TR/TE of 3200 ms/565 ms.
Area of acquisition	dHCP + HCP-YA: Whole brain

Diffusion MRI ☐ Used ☒ Not used

Preprocessing

Preprocessing software	dHCP: The structural and functional images were preprocessed using bespoke pipelines developed as part of the Developing Human Connectome Project. These have been previously published (structural - https://doi.org/10.1016/j.neuroimage.2018.01.054; functional - https://doi.org/10.1016/j.neuroimage.2020.117303) and are freely available (see above in "Software and Code").<br>HCP-YA: We used minimally preprocessed structural data, which is described in full by Glasser et al. (2013). Briefly, the PreFreeSurfer pipeline involves: correcting MR gradient nonlinearity-induced distortions; brain extraction; image intensity bias correction using the method described in Glasser et al. (2011); and correcting readout distortion. The distortion- and bias-corrected structural volumes were then passed through the Freesurfer pipeline which is a bespoke version of the FreeSurfer recon-all command that adjusts placement of the pial surface by exploiting intensity differences at the pial-cerebrospinal fluid boundary between T1w and T2w images. This modification ensures that lightly myelinated cortical grey matter is not artefactually excluded
Normalization	dHCP: All cortical surfaces were registered to a single 40-week left-right symmetric surface atlas, via template-to-template registration. The registration/normalisation process utilised multimodal surface matching, which is a freely available software that performs non-linear surface-based registration using higher order smoothness constraints.<br>HCP-YA: cortical surface were registered to the HCP MSMSulc template using multimodal surface matching.
Normalization template	dHCP: This is a new cortical surface atlas, and therefore subjects registered to this atlas are in dhcpSym40 space.<br>HCP-YA: HCP MSMSulc space
Noise and artifact removal	dHCP: For resting-state fMRI, FSL's fMRI EDDY was used for susceptibility-distortion correction and FSL FIX was used to identified structured noise such as physiological artefacts from cardiac pulsation (https://doi.org/10.1016/j.neuroimage.2020.117303).<br>For structural MRI, images were bias and motion corrected (https://doi.org/10.1016/j.neuroimage.2018.01.054).
Volume censoring	dHCP: End-end censoring, but no scrubbing, was performed.

Statistical modeling & inference

Model type and settings	dHCP: Permutation testing of general linear model with cortical asymmetry as the outcome, and adjusting for covariates including GA at birth, PMA at scan, biological sex, birthweight Z-score, and hemispheric volume asymmetry<br>HCP-YA: Age at scan, biological sex and hemispheric volume asymmetry
Effect(s) tested	No tasks were used in this study. Six one-sample t-tests were used to investigate cortical asymmetry (including associations with biological sex and PMA) in the term neonates, (C1: left > right, C2: right > left, C3: increasing PMA, C4: decreasing PMA, C5: female > male, and C6: male > female), and two two-sample unpaired t-tests were used to investigate differences in asymmetry between term and preterm neonates at TEA (C1: term > preterm, and C2: preterm > term). Six one-sample t-tests were performed to investigate cortical structural asymmetry in adults (C1: left > right, C2: right > left, C3: female > male, and C4: male > female, C5: increasing age, and C6: decreasing age), and two two-sample unpaired t-tests were performed to investigate differences in asymmetries between neonates and adults (C1: neonates > adults, and C2: adults > neonates).

Specify type of analysis: ☒ Whole brain ☐ ROI-based ☐ Both

Statistic type for inference<br>(See Eklund et al. 2016)	This study performed statistical analyses on the cortical surface using threshold-free cluster enhancement (TFCE). TFCE was performed on the group average midthickness anatomical surface, with the default H (2.0) and E (0.6) parameters.
Correction	Family-wise error rate corrections were applied to p-values across image features and design contrasts. Unthresholded p value and raw asymmetry index maps are available at https://balsa.wustl.edu/study/2xrBN.

Models & analysis

n/a	Involved in the study
☒	☐ Functional and/or effective connectivity
☒	☐ Graph analysis
☒	☐ Multivariate modeling or predictive analysis
