# Supplementary material for: Structural and functional asymmetry of the neonatal cerebral cortex
Source: Nat Hum Behav. Author manuscript; Available in PMC 2026 Mar 21. (PMC7618908; doi:10.1038/s41562-023-01542-8)
Supplement: Supplementary Information [file EMS212917-supplement-Supplementary_Information.pdf]

Supplementary Information for

Structural and functional asymmetry of the neonatal cerebral cortex

Williams, L. Z. J., Fitzgibbon, S. P., Bozek, J., Winkler, A. M., Dimitrova, R., Poppe, T., Schuh, A., Makropoulos, A., Cupitt, J., O'Muircheartaigh, J., Duff, E. P., Cordero-Grande, L., Price, A. N., Hajnal, J. V., Rueckert, D., Smith, S. M., Edwards, A. D. & Robinson, E. C.

Corresponding authors: Logan Z. J. Williams.
E-mail: logan.williams@kcl.ac.uk; Emma C. Robinson.
E-mail: emma.robinson@kcl.ac.uk

This PDF file includes:

Supplementary Figures 1 to 13
Supplementary Information References

Table of contents

Supplementary Figure 1 Parcellated asymmetry indices for surface area and cortical thickness 3
Supplementary Figure 2 Symmetric group resting-state networks for the dHCP 4
Supplementary Figure 3 $-\log_{10}(p)$-value maps for the effect of postmenstrual age on the structural and functional asymmetries in the healthy term-born neonatal cortex at term-equivalent age 5
Supplementary Figure 4 Difference in median asymmetry indices (female - male) of structural and functional asymmetries across the healthy term-born neonatal cohort scanned at term-equivalent age 6
Supplementary Figure 5 $-\log_{10}(p)$-value maps for the effect of biological sex on the structural and functional asymmetries in the healthy term-born neonatal cortex at term-equivalent age 7
Supplementary Figure 6 Difference in median asymmetry indices of structural and functional asymmetries between term-born and preterm-born neonates (term - preterm) scanned at term-equivalent age 8
Supplementary Figure 7 $-\log_{10}(p)$-value maps for the effect of preterm birth on the structural and functional asymmetries in the neonatal cortex at term-equivalent age 9
Supplementary Figure 8 Effect of surface registration on intersubject alignment of cortical folding. 10
Supplementary Figure 9 White matter surface for each postmenstrual week of the dhcpSym template 11
Supplementary Figure 10 Sulcal depth template for each postmenstrual week of the dhcpSym atlas 12
Supplementary Figure 11 Cortical thickness template for each postmenstrual week of the dhcpSym atlas 13
Supplementary Figure 12 T1w/T2w ratio template for each postmenstrual week of the dhcpSym atlas 14
Supplementary Figure 13 Understanding the centre-surround pattern 15

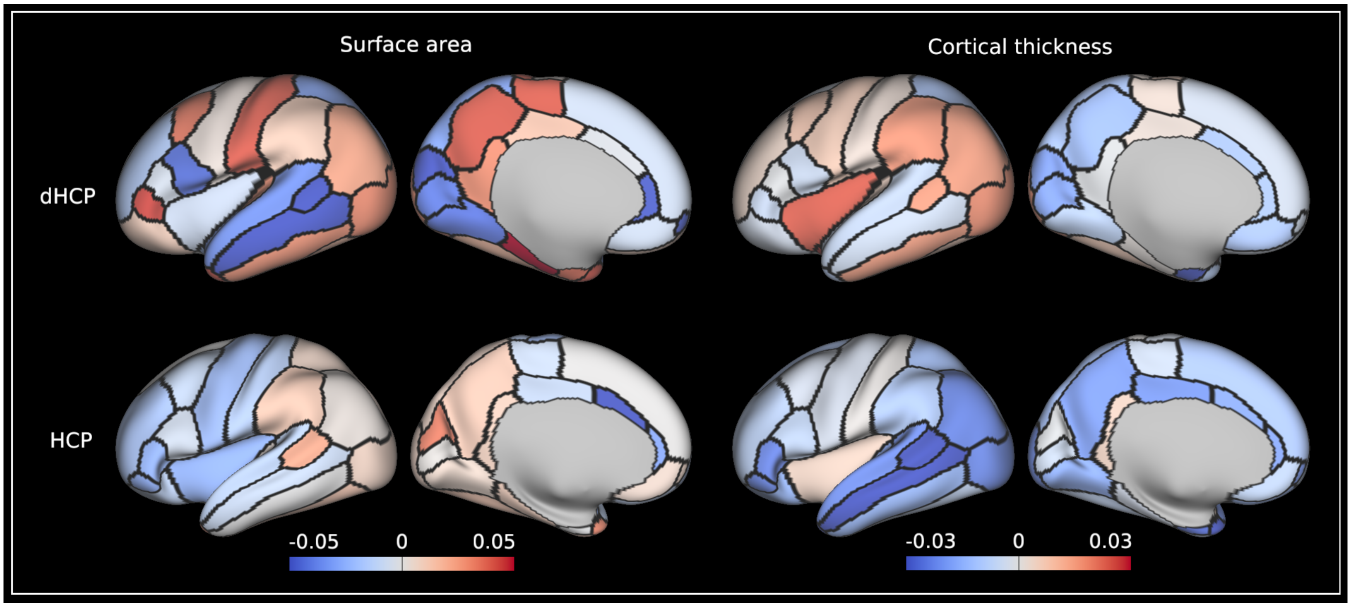

Supplementary Figure 1. Asymmetry indices of surface area and cortical thickness for the dHCP and HCP-YA cohorts, averaged (for the dHCP) within regions of the the M-CRIB-S atlas (neonatal equivalent of Desikan-Killiany atlas) (1, 2) and (for HCP-YA) the Desikan-Killiany atlas (3). Leftward asymmetry indices are color-coded red, and rightward asymmetry indices are color-coded blue. Data at https://balsa.wustl.edu/1pn1V.

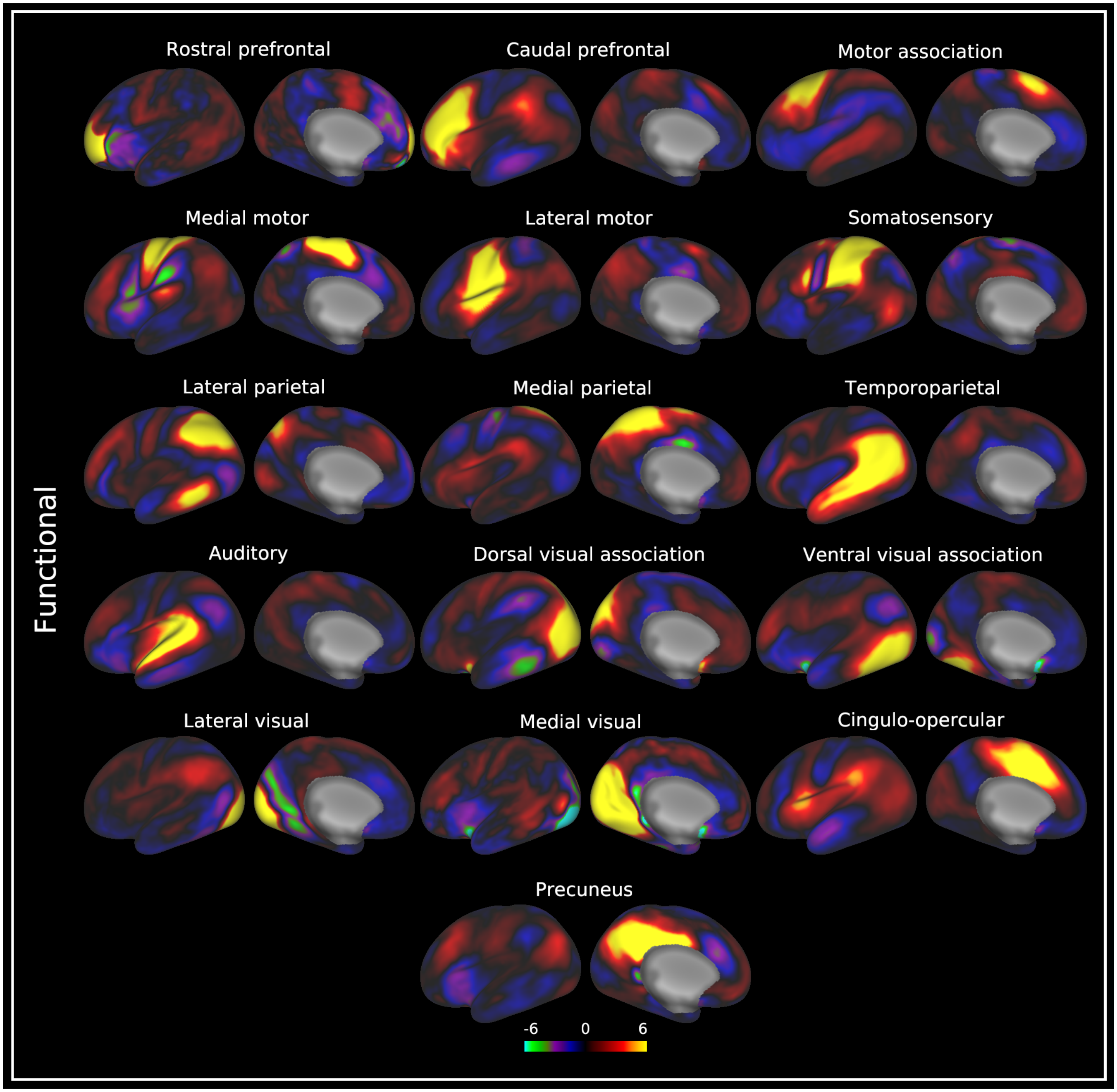

Supplementary Figure 2. Symmetric group resting-state networks for the dHCP. Resting-state network spatial maps visualised on very inflated 40-week PMA left hemispheric surface. Only the left hemisphere is shown for illustrative purposes. Data at https://balsa.wustl.edu/5B3M3.

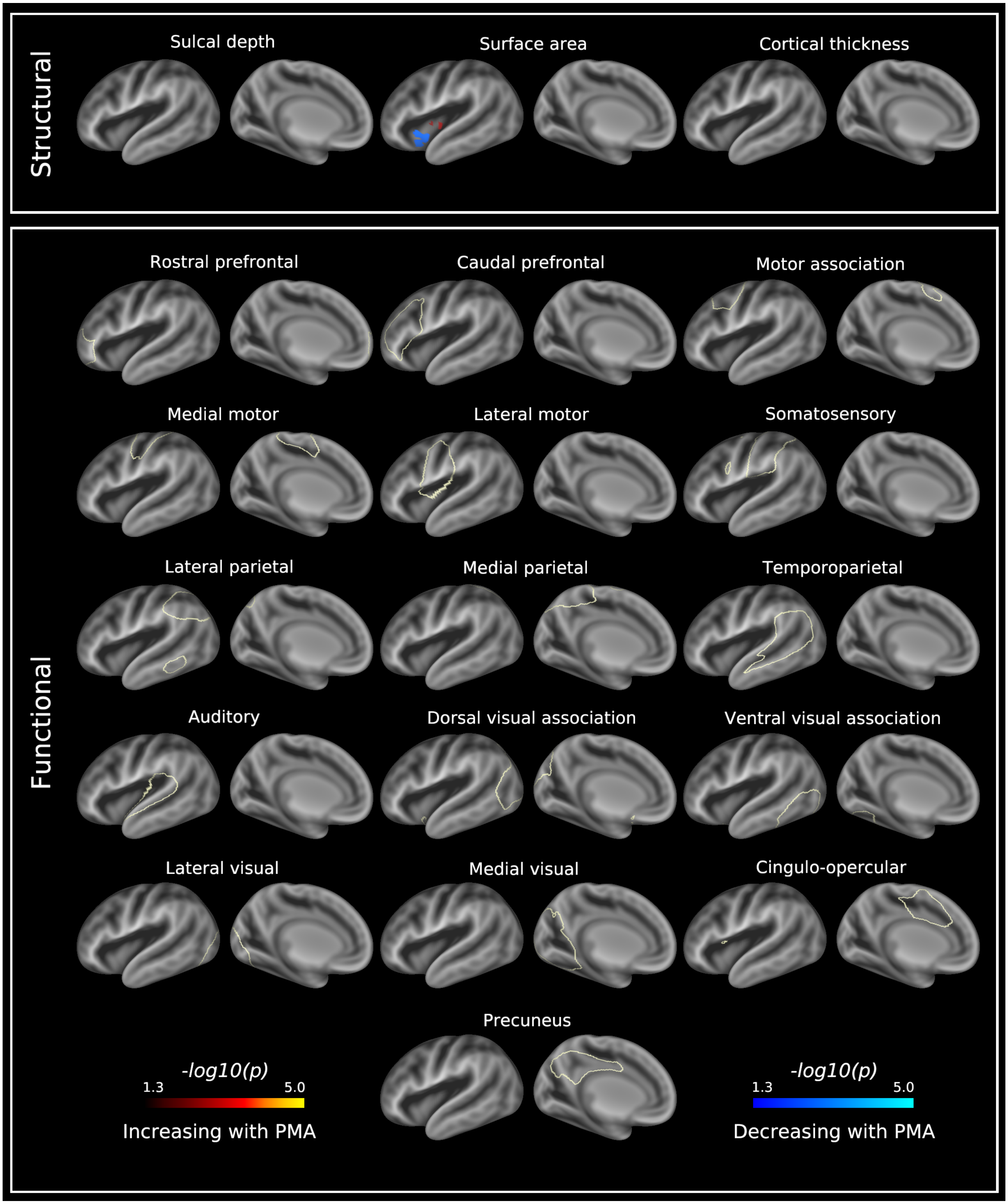

Supplementary Figure 3. $-\log_{10}(p)$-value maps for the effect of postmenstrual age on the structural and functional asymmetries in the healthy term-born neonatal cortex at term-equivalent age. Leftward asymmetries are represented by the red-yellow colour scale and rightward asymmetries by the blue-light blue colour scale. Significantly asymmetric regions are visualised on a very inflated 40-week PMA left hemispheric surface, and are overlaid on a 40-week PMA sulcal depth template (grey scale colour scheme). Off-white lines surrounding the functional asymmetries represent the mask used to threshold single subject asymmetry maps (see Methods: Generating Asymmetry Maps). Data at https://balsa.wustl.edu/n8Gvj.

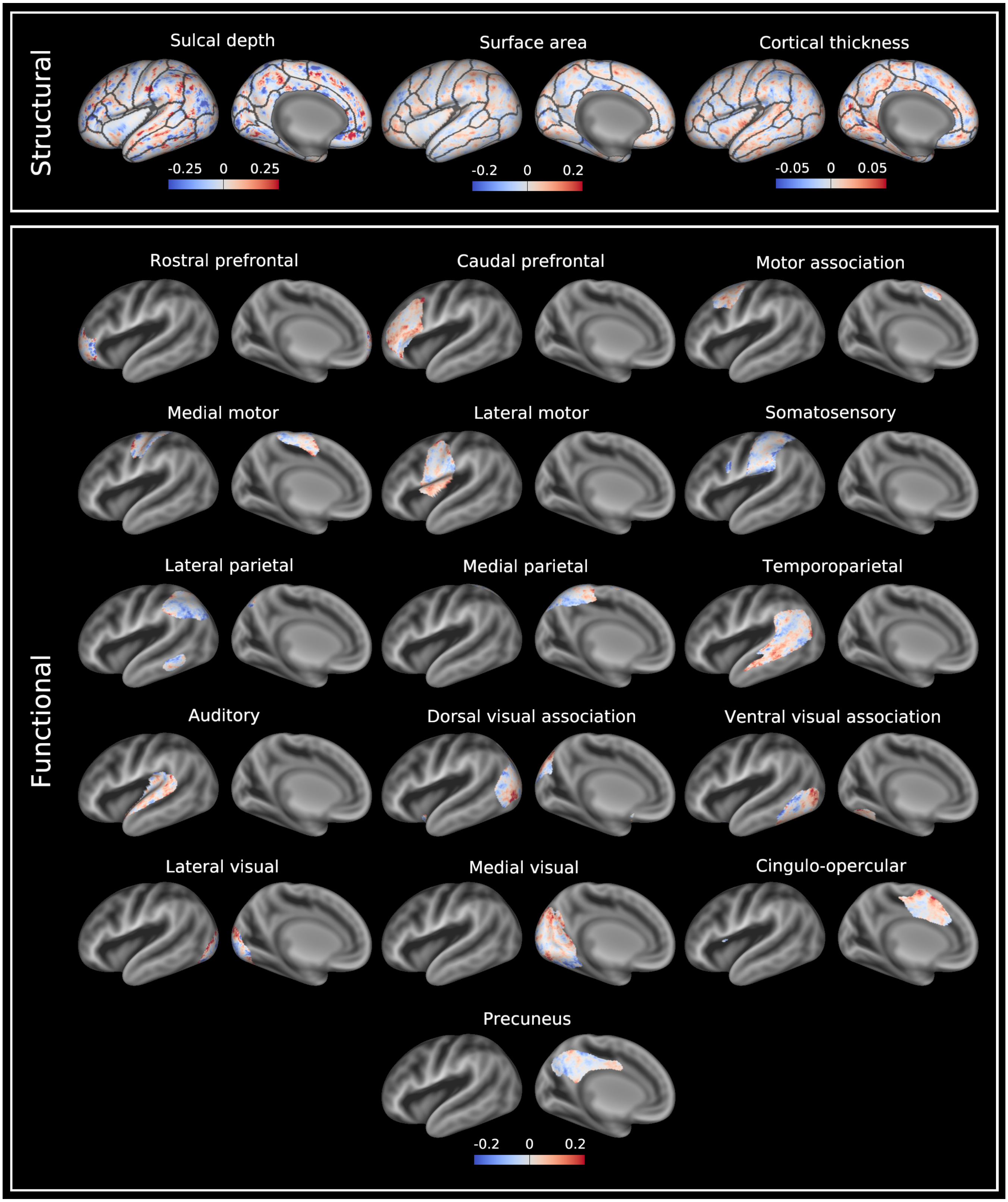

Supplementary Figure 4. Difference in median asymmetry indices (female - male) of structural and functional asymmetries across the healthy term-born neonatal cohort scanned at term-equivalent age. Leftward asymmetries are color-coded red, and rightward asymmetries color-coded blue. Asymmetry indices are visualised on a very inflated 40-week PMA left hemispheric surface, and are overlaid on a 40-week PMA sulcal depth template (grey scale colour scheme). Anatomical regions of interest from a neonatal version of the Desikan-Killiany atlas (M-CRIB-S) (1, 2) are overlaid on the structural asymmetries for reference. Data at https://balsa.wustl.edu/gmgnv

.

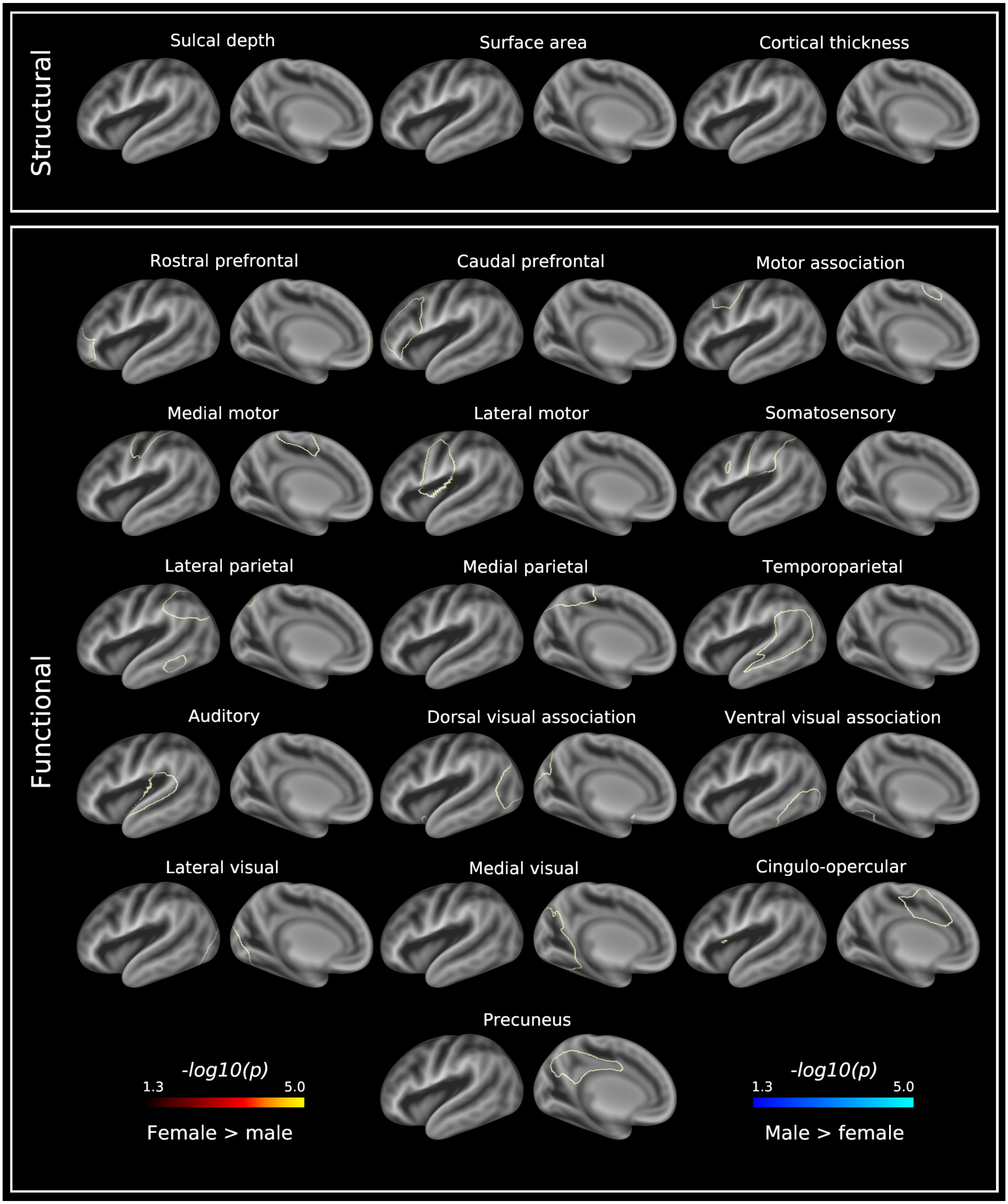

Supplementary Figure 5. $-\log_{10}(p)$-value maps for the effect of biological sex on the structural and functional asymmetries in the healthy term-born neonatal cortex at term-equivalent age. Leftward asymmetries are represented by the red-yellow colour scale and rightward asymmetries by the blue-light blue colour scale. Significantly asymmetric regions are visualised on a very inflated 40-week PMA left hemispheric surface, and are overlaid on a 40-week PMA sulcal depth template (grey scale colour scheme). Off-white lines surrounding the functional asymmetries represent the mask used to threshold single subject asymmetry maps (see Methods: Generating Asymmetry Maps). Data at https://balsa.wustl.edu/M9jX2.

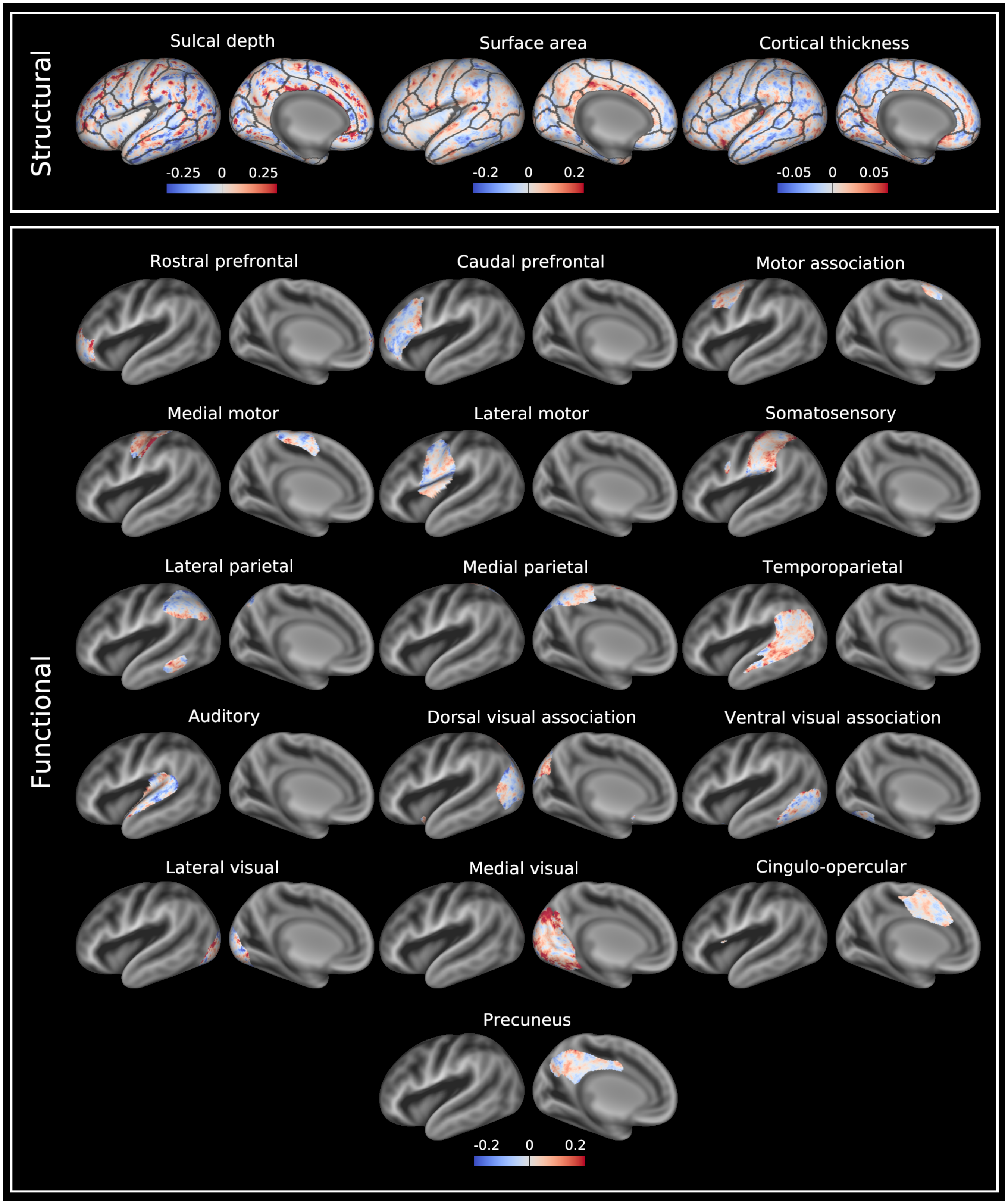

Supplementary Figure 6. Difference in median asymmetry indices of structural and functional asymmetries between term-born and preterm-born neonates (term - preterm) scanned at term-equivalent age. Leftward asymmetries are color-coded red, and rightward asymmetries color-coded blue. Asymmetry indices are visualised on a very inflated 40-week PMA left hemispheric surface, and are overlaid on a 40-week PMA sulcal depth template (grey scale colour scheme). Anatomical regions of interest from a neonatal version of the Desikan-Killiany atlas (M-CRIB-S) (1, 2) are overlaid on the structural asymmetries for reference.Data at https://balsa.wustl.edu/Bg8Xm.

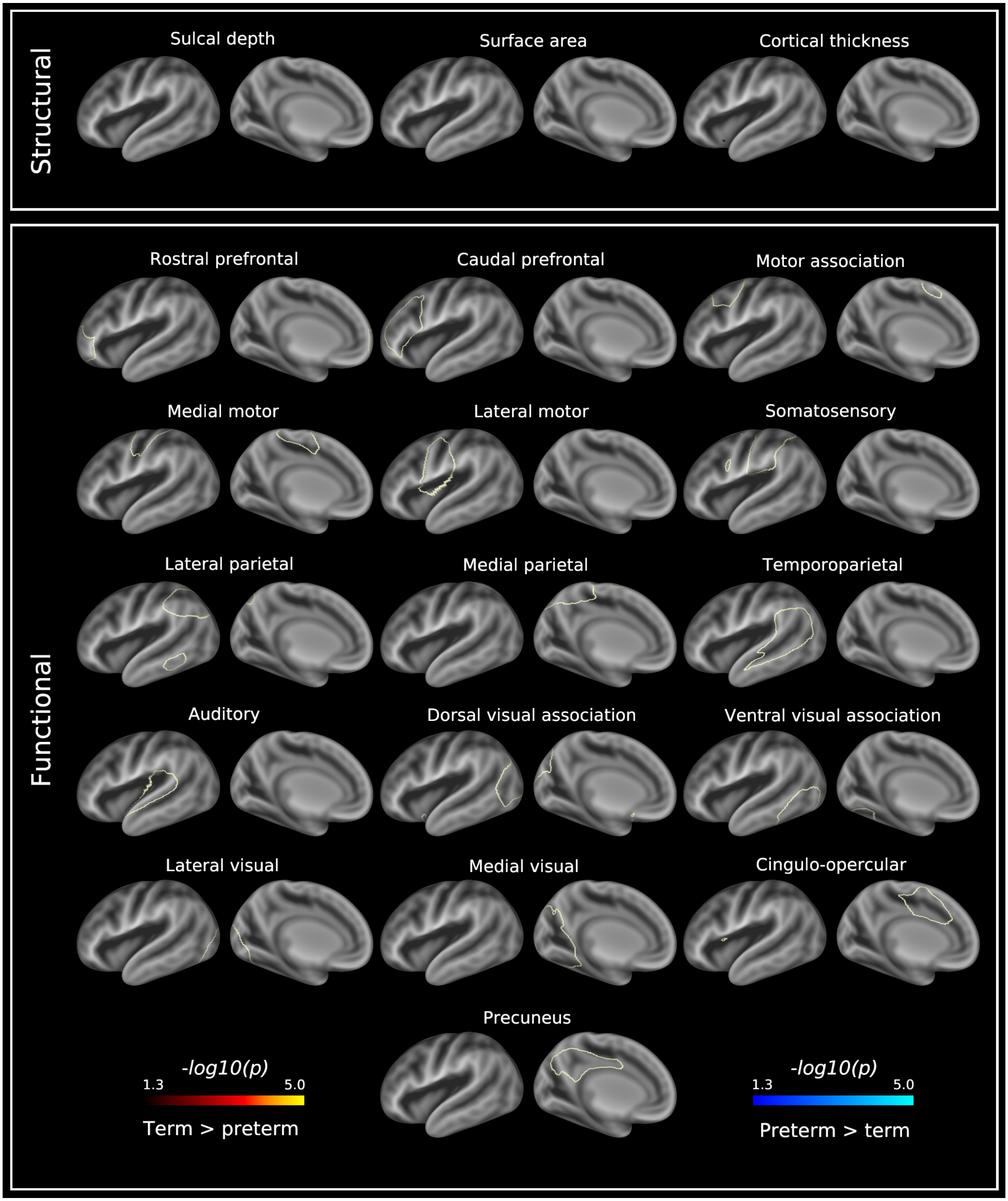

Supplementary Figure 7. $-\log_{10}(p)$-value maps for the effect of preterm birth on the structural and functional asymmetries in the neonatal cortex at term-equivalent age. Leftward asymmetries are represented by the red-yellow colour scale and rightward asymmetries by the blue-light blue colour scale. Significantly asymmetric regions are visualised on a very inflated 40-week PMA left hemispheric surface, and are overlaid on a 40-week PMA sulcal depth template (grey scale colour scheme). Off-white lines surrounding the functional asymmetries represent the mask used to threshold single subject asymmetry maps (see Methods: Generating Asymmetry Maps). Data at https://balsa.wustl.edu/l7gq0.

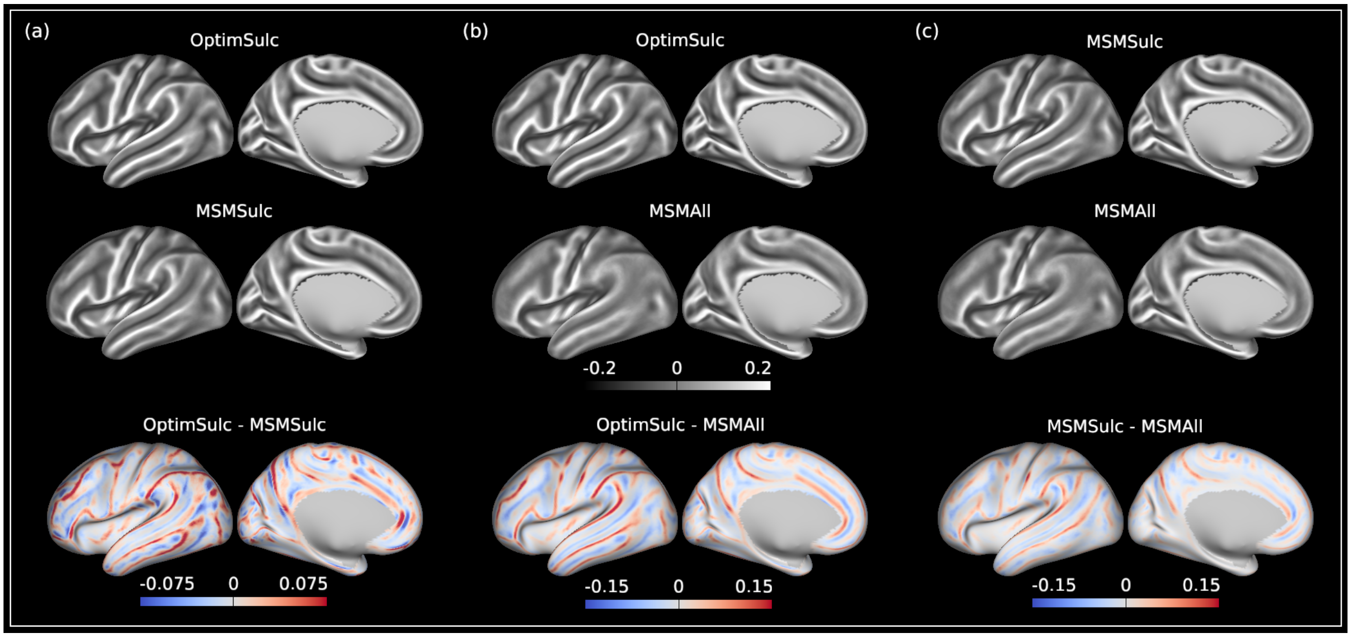

Supplementary Figure 8. Effect of surface registration on intersubject alignment of cortical folding. (a) Top row: median curvature maps across 1110 HCP-YA subjects after registration optimised for sulcal depth (OptimSulc); middle row: median curvature maps across 1110 HCP-YA subjects after MSMSulc registration; bottom row: difference in median curvature maps after OptimSulc and MSMSulc registration (OptimSulc - MSMSulc). (b) Top row: median curvature maps across 1110 HCP-YA subjects after OptimSulc registration; middle row: median curvature maps across 1096 HCP-YA subjects after MSMAll registration; bottom row: difference in median curvature maps after OptimSulc and MSMAll registration (OptimSulc - MSMAll). (c) Top row: median curvature maps across 1110 HCP-YA subjects after MSMSulc registration; middle row: median curvature maps across 1096 HCP-YA subjects after MSMAll registration; bottom row: difference in median curvature maps after MSMSulc and MSMAll registration (MSMSulc - MSMAll). Median curvature maps are visualised on very inflated left hemispheric surfaces for illustrative purposes. Data at https://balsa.wustl.edu/Bg0jv.

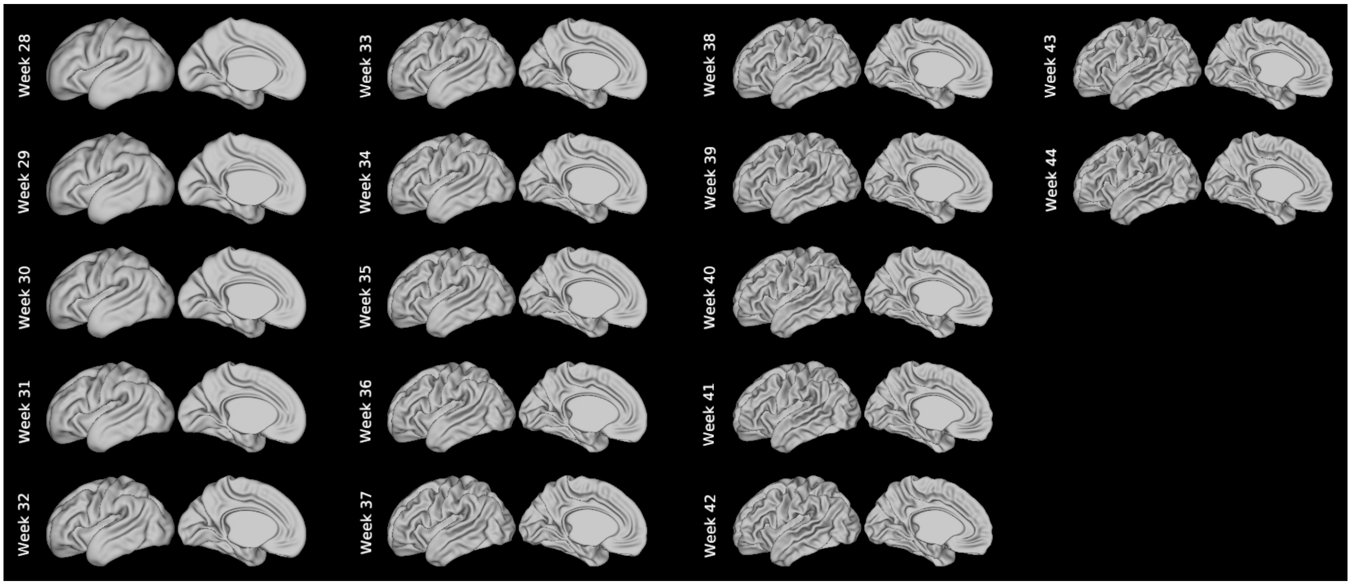

Supplementary Figure 9. White matter surface for each postmenstrual week of the dhcpSym template. Only the left hemisphere is shown for visualisation purposes. Data at https://balsa.wustl.edu/qx9ZB.

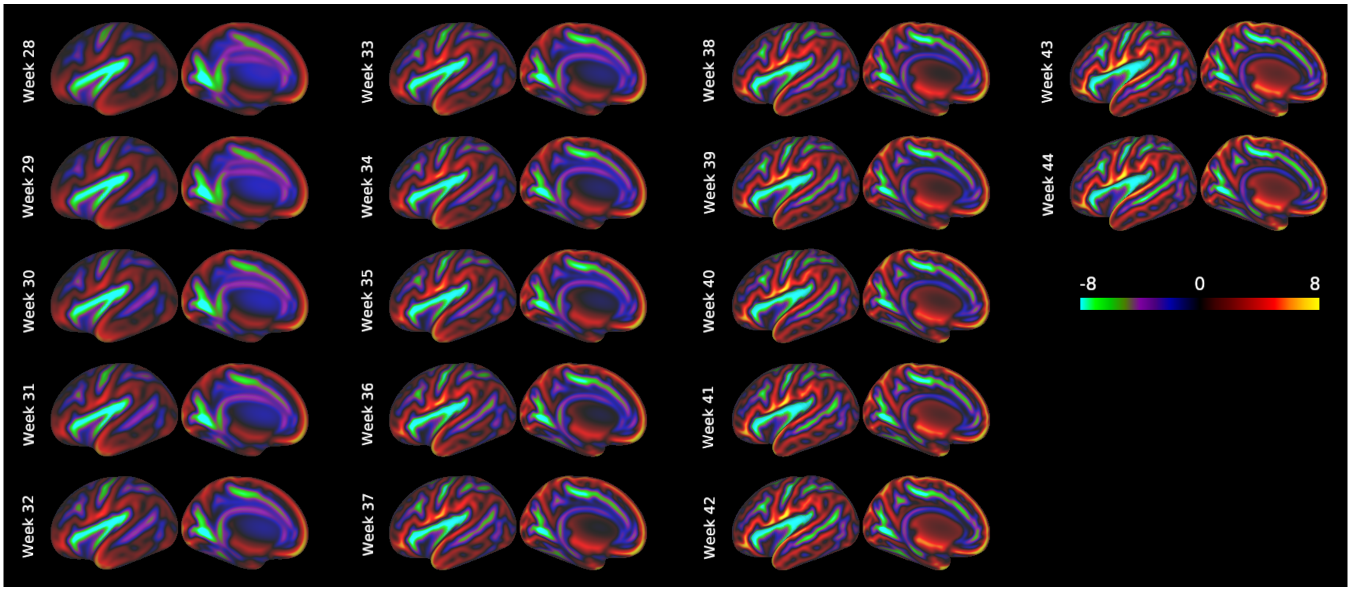

Supplementary Figure 10. Sulcal depth template for each postmenstrual week of the dhcpSym atlas. Sulcal depth templates are overlaid on a very inflated left hemispheric surface for visualisation purposes. Data at https://balsa.wustl.edu/jNgB2.

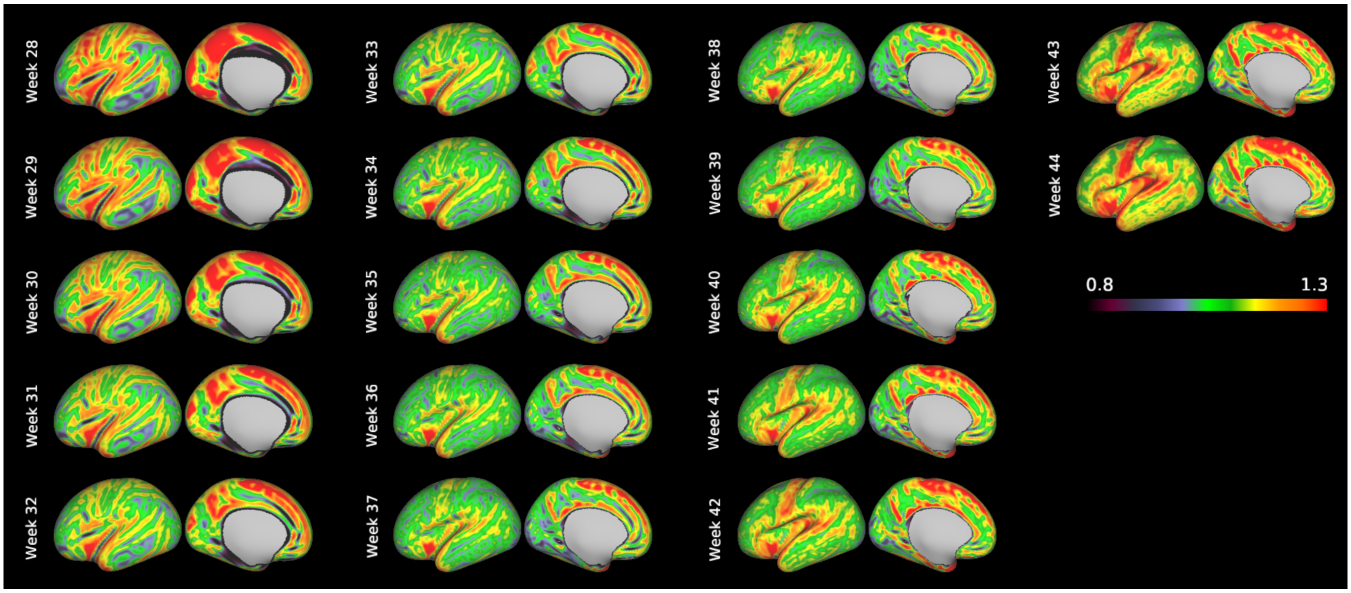

Supplementary Figure 11. Cortical thickness template for each postmenstrual week of the dhcpSym atlas. Cortical thickness templates are overlaid on a very inflated left hemispheric surface for visualisation purposes. Data at https://balsa.wustl.edu/w8MkL.

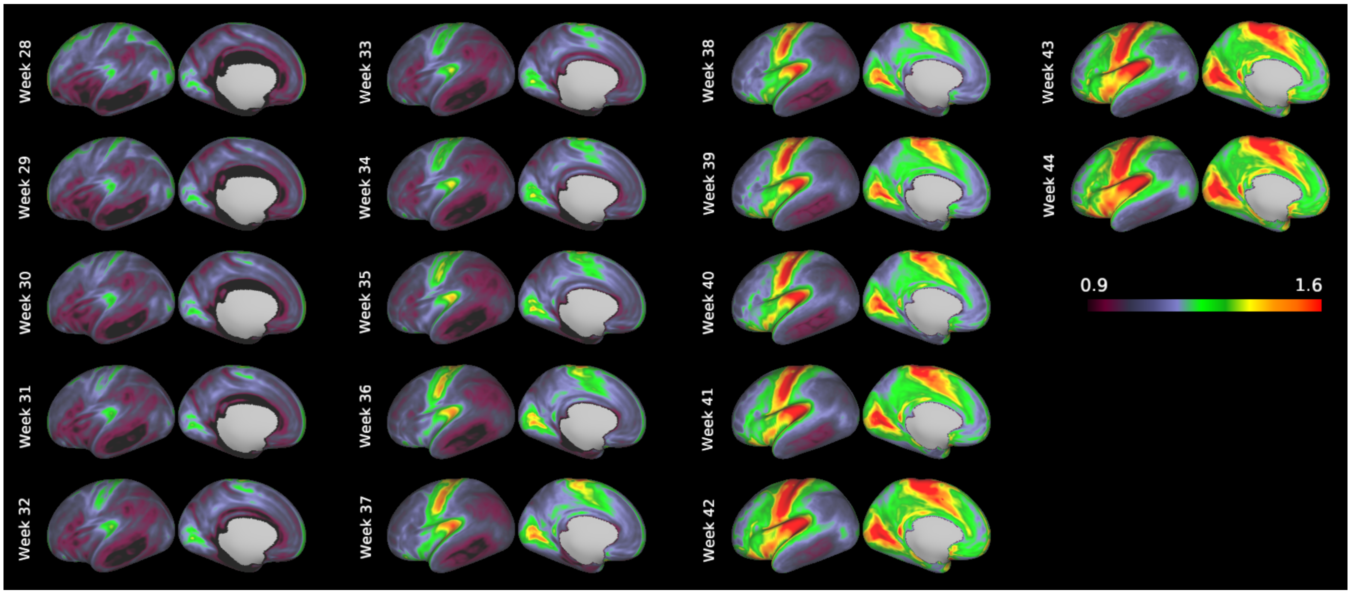

Supplementary Figure 12. T1w/T2w ratio template for each postmenstrual week of the dhcpSym atlas. T1w/T2w ratio templates are overlaid on a very inflated left hemispheric surface for visualisation purposes. Data at https://balsa.wustl.edu/40Pgj.

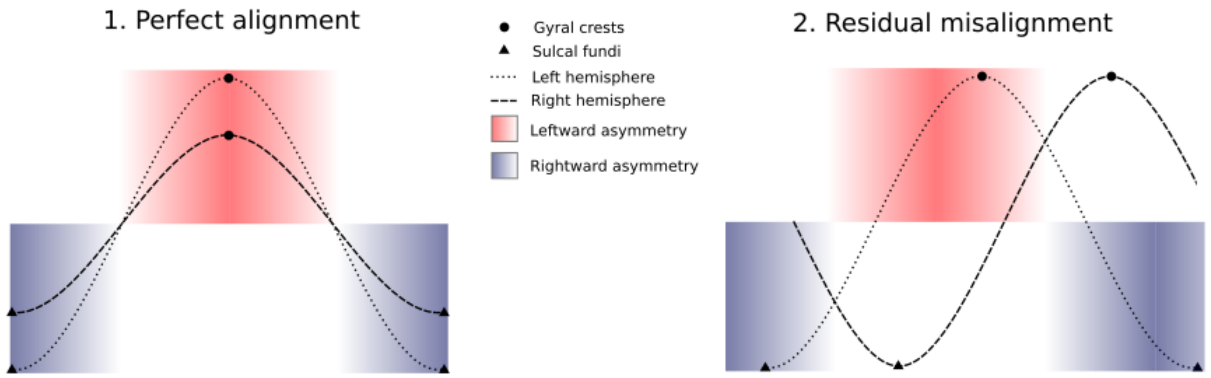

Supplementary Figure 13. Understanding the centre-surround pattern. Schematic of left and right cortical folds in two registration scenarios: 1. Perfect alignment, where the location of the gyral crests/sulcal fundi/inflection points (transition between gyrus and sulcus) correspond across left and right hemispheres. In this instance, the left cortical fold has higher amplitude (greater gyral height and lower sulcal depth), which creates a centre of leftward asymmetry flanked by areas of rightward asymmetry. In the other scenario, 2. Residual misalignment, the same centre surround pattern has been produced despite there being no true differences in sulcal depth asymmetry.

References

1. B Alexander, et al., A new neonatal cortical and subcortical brain atlas: the melbourne children's regional infant brain (m-crib) atlas. *NeuroImage* **147**, 841–851 (2017).
2. CL Adamson, et al., Parcellation of the neonatal cortex using surface-based melbourne children's regional infant brain atlases (m-crib-s). *Sci. reports* **10**, 1–11 (2020).
3. RS Desikan, et al., An automated labeling system for subdividing the human cerebral cortex on mri scans into gyral based regions of interest. *Neuroimage* **31**, 968–980 (2006).
